# Endometrial osseous metaplasia complicated by secondary infertility: a case report

**DOI:** 10.11604/pamj.2021.40.61.30691

**Published:** 2021-09-28

**Authors:** Olalekan Abisola Ajayi, Olufemi Oladipupo Adebawojo, Victor Chimezie Okebalama

**Affiliations:** 1 Department of Histopathology, Babcock University Teaching Hospital, Ilisan-Remo, Ogun State, Nigeria,; 2 Department of Obstetrics and Gynecology, Babcock University Teaching Hospital, Ilisan-Remo, Ogun State, Nigeria

**Keywords:** Endometrial osseous metaplasia, secondary infertility, hysteroscopy, case report

## Abstract

Endometrial osseous metaplasia is a rare condition in which there is abnormal presence of bone in the endometrium. There is paucity of reported cases of this pathological condition in Africa and it is usually overlooked as a cause of infertility. Pathogenesis is not well understood but mostly occurs following pregnancy. Pathology may be suspected on ultrasound scan where a linear echogenic substance is seen in the endometrium but diagnosis is confirmed by and treated with hysteroscopy. We present a case of a 43-year-old woman with 2 previous miscarriages who presented with secondary infertility. She had an ultrasound scan done which revealed features suggestive of an intra-uterine copper device. She subsequently had hysteroscopy and a bone-like foreign body was found in the endometrium which was removed with the aid of a grasper and later sent for histopathological evaluation for which a subsequent diagnosis of endometrial osseous metaplasia was made. Indeed, endometrial intraosseous metaplasia should be considered an important differential cause of secondary infertility especially in patients with history of previous miscarriage. Confirmatory diagnosis can be made through hysteroscopy and/or histopathologically although the former is now used.

## Introduction

Endometrial osseous metaplasia is an infrequent entity involving the presence of mature or immature bone in the endometrium [1]. The incidence is about 3 in 10,000 and most women are of the reproductive age [2,3]. Majority of the reported cases, the women presented with infertility. However, they may present with other gynecological symptoms. There seems to be an association with previous spontaneous or induced abortion and the disease entity [4-6]. Different theories have been postulated to describe the pathogenesis of the endometrial osseous metaplasia, but metaplasia of the endometrial stromal cells, particularly fibroblasts, into bone forming osteoblasts is the most accepted [4,7]. Although pelvic ultrasound scan can aid in the diagnosis, the gold standard for treatment is hysteroscopy [6,8-10]. In most reported cases, fertility is restored after treatment. Here, we present a case report of 43-year-old woman with secondary infertility and was managed hysteroscopically.

## Patient and observation

**Patient information:** a 43-year-old woman presented to our gynecological clinic for hysteroscopy in the month of March, 2021 on referral basis as part of her evaluation for secondary infertility. She was being managed for infertility of 2 years´ duration. She has had 2 previous miscarriages. First was a voluntary termination of pregnancy 25 years ago at 12 weeks of gestation using manual vacuum aspiration. She also had a spontaneous termination of pregnancy a year ago at 8 weeks´ gestation. Retained products of conception were evacuated using manual vacuum aspiration. In both cases, there were no immediate post abortal complications. She had a regular menstrual period. The family and psychosocial history revealed that she is married in a monogamous setting. Her husband is 45-year-old and they both have tertiary level of education. She does not smoke or take alcohol. Also, she does not use recreational drugs. There was no family history of infertility known to her. However, there was history of galactorrhea and she was been treated with bromocriptine.

**Clinical findings:** at presentation to the obstetrics and gynecology clinic, physical examination showed a young woman, not ill looking or pale, anicteric, not dehydrated with no palpable peripheral lymph node and no pedal oedema. On speculum examination, a healthy looking cervix and vaginal walls were seen. Furthermore, on bimanual examination, normal-sized anteverted uterus with no adnexal mass or tenderness were found. Additionally, there was no cervical excitation tenderness.

**Diagnostic assessment:** she had transvaginal ultrasonography and hysterosalpingography sequentially 3 days post her first day of presentation while her husband´s seminal fluid was collected for analysis and culture 1 week later. The transvaginal ultrasound scan done revealed a linear echogenic structure with the median aspect of the mass in the posterior endometrium. Also, the performed hysterosalpingography showed bilateral tubal patency. In addition, the husband´s seminal fluid analysis and culture results which became available 3 days following collection showed normal findings. Indeed, there were no diagnostic challenges.

**Diagnosis:** a presumptive diagnosis of malpositioned intrauterine device was made following transvaginal ultrasonography findings.

**Therapeutic interventions:** furthermore, 7 days following hysterosalpingography, hysteroscopy was performed on her using saline as a distension medium. It revealed a spicule of bone-like foreign body about 2cm long in the posterior wall of the uterus. The other parts of the endometrial cavity looked grossly normal.

**Figure 1:**
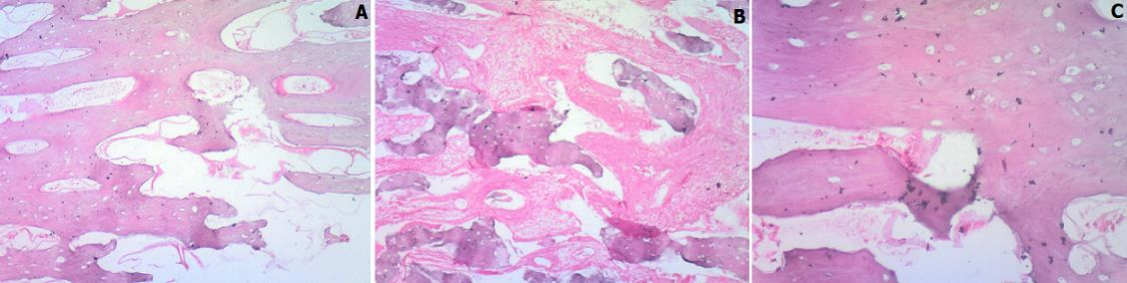
photomicrograph showing mature bony trabeculae with non-hemopoietic bone marrow within a bland fibrocollagenous stroma and occasional chronic inflammatory cells: A) H & E X 40; B) H & E X 4; C) H & E X 100

**Follow-up and outcome of interventions:** patient was clinically stable post procedure. There were no adverse effects. Also, the specimen discovered on hysteroscopy was subsequently sent to the laboratory for histology on same day of the procedure. Additionally, the histology result which became available 14 days from the day of request revealed a hard cord-like greyish white tissue measuring 1.5 x 0.2 x 0.2cm in the antero-posterior diameter. Microscopically, section showed mature bony trabeculae with non-hemopoietic bone marrow within a bland fibrocollagenous stroma. Occasional chronic inflammatory cells were also seen (Figure 1A, B, C). The diagnosis of endometrial osseous metaplasia was made. She was subsequently referred back to her primary place of care 24 days from her first day of presentation.

**Patient perspective:** I had a reassuring experience receiving treatments at Babcock University Teaching Hospital. Treatments and laboratory test were prompt and my condition was explained to me in language I understood. I understood that although my condition is associated with inability to conceive, there may be other reasons why there is delay. I am hopeful of getting pregnant soon. Thank you Babcock.

**Informed consent:** written consent was obtained from the patient to publish images and clinical information relating to the case in any medical publication of choice for the purpose of knowledge sharing and educating the public. The patient was made to understand that no identifying information will be collected or published.

## Discussion

Endometrial osseous metaplasia is an endogenous non-neoplastic pathological disorder [11]. It has also been termed endometrial ossification, ectopic intrauterine bone and heterotopic intrauterine bone. It can be found in the ovaries, cervix and vagina [9]. Endometrial osseous metaplasia is a rare clinical entity with an incidence of 3/10,000 and less than 100 cases has been reported in literature [12]. Okohue *et al*. had reported an incidence of 0.26% in 1002 hysteroscopies in a study done in Nigeria [13]. Even though women in the reproductive age group are mostly affected, a case has been reported in a post-menopausal woman [7, 9]. Presence of bony tissue has been linked to abortion since 1923, with more than 80% of affected women said to have an antecedent history of first trimester abortion, either spontaneous or induced [8]. However, it has also been reported in women with no history of prior pregnancy [14]. In line with literature, index patient was in the reproductive age group, had a bony-like foreign body discovered at the posterior aspect of her uterus during hysteroscopy which was confirmed histologically to be a bone tissue. Additionally, prior to presentation, she has had two previous miscarriages, one induced and the other spontaneous, while both occurred within the first trimester. The time gap between previous abortion and diagnosis of endometrial osseous metaplasia varies between 8 weeks and 14 years in most cases, though an interval of 37 years has been reported [3]. The time differences in our patient were 25 years and one year, respectively, for the two previous miscarriages. However, delayed diagnosis may be due to late presentation for the management of infertility.

Patient typically presents with secondary infertility from cases reported but may also present with oligomenorrhea, menorrhagia, dyspareunia, pelvic pain and vaginal discharge [6,10]. They may also be asymptomatic [5]. In our case, our patient presented with infertility. Indeed, presence of bony tissue in the endometrium may act as an intrauterine contraceptive device elevating the levels of prostaglandins thus preventing implantation of the blastocyst [15]. However, our patient´s infertility history may not only be due to endometrial osseous metaplasia, as her age and galactorrhea may also be contributing factors. Theories for the development of heterotopic bone in the endometrium are controversial, but the most favorable mechanism is osseous metaplasia from multipotential stromal cells, usually fibroblasts, which subsequently become osteoblasts [3]. Other suggested mechanisms include: retention of fetal bones that secondarily promote osteogenesis in the surrounding endometrium [8] which may be the mechanism in index case considering she had an induced abortion at 13 weeks of gestation when osteogenesis would have started, continuous and strong endometrial estrogenic stimulation, implantation of embryonic parts without pre-existing bone after abortions at an early stage; dystrophic calcification of retained and necrotic tissues, usually after an abortion [8]. Additionally, chronic endometrial inflammation such as endometritis or pyometra can also play a role by stimulating mesenchymal cells which have the capacity to undergo metaplasia and differentiate into chondroblast or osteoblast [8,9]. Furthermore, chronic inflammation may stimulate mononuclear phagocytes to release tumor necrosis factor and superoxide radicals and this will lead to long-lasting insult to the pluripotent stromal cells transforming them to osteoblast in endometrium deficient of superoxide dismutase [8]. In addition, a case of endometrial osseous metaplasia due to metabolic disorders such as hypercalcemia, hypervitaminosis D or hyperphosphatemia has been reported [2].

The differential diagnosis of endometrial osseous metaplasia includes intra-uterine contraceptive device, malignant mixed mullerian tumour, endometrial tuberculosis and retained fetal bone in the uterus [1]. The incidence of retained fetal bone in Nigeria is 0.15% [16]. Although, the presence of retained bone in the uterus will have similar history and symptom, absence of endochondral ossification and surrounding tissue reaction may differentiate it from endometrial osseous metaplasia [8]. Even though in our case there was no endochondral ossification, but there was presence of non-hemopoietic bone marrow which has been previously reported [17]. Additionally, ultrasound scan plays an important role in the diagnosis of endometrial osseous metaplasia. Here, presence of hyperechogenic pattern in the endometrium is suggestive [8]. In our case, a linear echogenic structure was seen in the endometrium. Modalities of treatment include dilatation and curettage, hysterectomy and hysteroscopy. However, hysteroscopy is being considered the gold standard for treatment [5,9,10]. Furthermore, in cases of widespread ossification of the endometrium, hysteroscopy can be performed under ultrasound guidance or laparoscopy to prevent perforation [18]. Usually after treatment, fertility returns in most reported cases [3,8] but this has not been confirmed in index patient as she has been referred back to her primary place of care.

## Conclusion

Endometrial osseous metaplasia is an uncommon but treatable cause of secondary infertility. Physicians must have a high index of suspicion for diagnosis to be made early. Hysteroscopy is now the method of choice for diagnosis and treatment.
